# Finite Element Implementation of Delta-P1 Model for Simulation of Photothermal Cancer Therapy in Heterogeneous Tissues

**DOI:** 10.3390/nano16040279

**Published:** 2026-02-23

**Authors:** Roberto C. Gómez-Araque, Carlos A. Bustamante-Chaverra, Raúl A. Valencia-Cardona, Whady F. Flórez-Escobar

**Affiliations:** School of Engineering, Universidad Pontifica Bolivariana, Medellín 050031, Colombia; roberto.gomeza@upb.edu.co (R.C.G.-A.); carlos.bustamante@upb.edu.co (C.A.B.-C.); whady.florez@upb.edu.co (W.F.F.-E.)

**Keywords:** Delta-P1 model, photothermal therapy, biological tissues, finite element method, gold nanoparticles

## Abstract

Photothermal therapy (PTT) is an emerging non-invasive treatment for cancer, offering targeted, localized therapy with minimal side effects. Its growing significance lies in its ability to precisely heat and destroy tumor cells while sparing surrounding healthy tissue. This study aimed to validate the δP1 approximation for simulating light propagation and thermal effects in biological tissues, particularly for photothermal therapy (PTT) applications. The model is applied to various scenarios, including homogeneous and heterogeneous tissue geometries with different optical properties and nanoparticle concentrations. The results are compared with analytical solutions, Monte Carlo results and experimental data to assess model accuracy. The δP1 approximation demonstrates superior performance compared to Beer–Lambert and Standard diffusion models, accurately predicting temperature distributions and capturing the influence of heterogeneous geometries. These findings highlight the potential of the δP1 model to significantly advance the field of PTT by providing reliable predictions for treatment planning and optimization.

## 1. Introduction

Cancer remains a leading cause of death worldwide, with approximately 10 million deaths attributed to the disease in 2020 [1]. While traditional therapies such as chemotherapy, radiotherapy, and surgery have limitations, emerging therapies like photothermal therapy (PTT) offer a promising non-invasive and targeted approach [2]. PTT involves irradiation of tissue containing cancerous cells with laser electromagnetic waves, leading to localized hyperthermia and subsequent cell death by apoptosis [3]. Metallic nanoparticles, particularly gold nanoparticles, are well suited for this therapy because of their surface plasmon resonance property. Upon excitation at a specific wavelength, these nanoparticles efficiently convert light into heat through electron–phonon interactions [3]. Numerical modeling is a valuable tool for predicting tissue response and temperature distribution during PTT [4]. This phenomenon couples two key aspects: heat transport and radiative transport [5]. In the first component, the Pennes bioheat model is a well-established tool to predict the distribution of temperature in living tissue, taking into account biological heat sources such as metabolic activity and blood perfusion [6]. The heat generated by nanoparticles is frequently modeled as a source term in the bioheat equation as a function in terms of the absorption coefficient and the fluence rate [7]. The fluence rate is modeled through approximations of the Radiative Transfer Equation (RTE), as its direct numerical solution poses significant computational challenges [8]. Previous studies have shown that the choice of the radiative transport model that approximates the RTE significantly affects temperature predictions [9,10]. Common models used in PTT to approximate RTE include the Beer–Lambert law (BL), Monte Carlo (MC) and the standard diffusion approximation (SDA) [7,10,11,12]. Although SDA has been widely used to approximate the fluence rate and nanoparticle-generated heat in highly scattering media such as tissues in the near-infrared spectrum [9,10], its precision decreases when the scattering coefficient is not significantly larger than the absorption coefficient [9,13]. MC simulations, which provide a probabilistic solution to the RTE, are preferred for biological tissues due to their superior accuracy compared to deterministic methods like SDA, particularly in terms of handling a broader range of optical properties, refractive index variations, and heterogeneous tissue geometries [14]. However, its computational cost limits its use as a forward model [13]. To address the limitations of SDA while preserving computational efficiency, alternative approximations have been explored, such as higher-order spherical harmonic expansions [15]. Among these, the δP1 model stands out for its ability to maintain accuracy across a wider range of optical properties and refractive index mismatches, particularly in tissues with high absorption coefficients, such as those containing nanoparticles [16]. The authors have previously reported a finite element implementation of the δP1 model in two-dimensional geometries [17]. Previous studies in nanoparticle-assisted photothermal therapy, such as Bruno et al. [18], showed that the classical P1 approximation can reproduce temperature fields comparable to higher-fidelity radiative solvers when applied to idealized one-dimensional slab geometries with isotropic scattering. However, the P1 model is known to lose accuracy in regimes where absorption and forward scattering compete, and its applicability is largely restricted to simplified geometries and narrow optical conditions [19]. Recent work by Maurente and de Sousa [20] systematically analyzed the impact of anisotropic scattering on photothermal therapy modeling, demonstrating that classical diffusion-based and P1 formulations may lose accuracy under optical regimes characterized by strong forward scattering and heterogeneous media, when compared to Monte Carlo benchmarks. Their study highlights the need for radiative transport models that extend beyond standard P1 assumptions while remaining computationally efficient.

The proposed numerical framework, which is based on the δP1 model and Pennes’ bioheat equation, is flexible enough to be extended to the study of chemically activated therapies [21]. This can be achieved by incorporating additional reaction–diffusion energy source terms into the bioheat equation. Integrating terms that model reaction kinetics and the consumption of components such as oxygen would enable the model to more accurately predict the thermal and biological impact of secondary chemical reactions triggered after phototherapy. However, the full treatment and numerical validation of these multifaceted therapies is beyond the scope of this study and will be the focus of future research.

In this context, the present work provides a direct and complementary advancement by adopting the δP1 approximation, which explicitly separates collimated and diffuse radiation components and improves accuracy across a broader range of absorption–scattering ratios. The novelty of this study lies in the finite element implementation of δP1 for two-dimensional heterogeneous tissues, its coupling with transient bioheat transfer, and its systematic validation against analytical solutions, Monte Carlo simulations, and experimental phantom data. Established bioheat formulations and optical property models are reused, while the radiative transport treatment, numerical framework, and validation scope constitute the main new contributions.

## 2. Materials and Methods

The fluence rate, described by the light transport phenomena, is as a heat source term for the subsequent thermal model, such as Pennes’ bioheat equation, to simulate heat distribution in tissue. The propagation of visible or infrared photons through a turbid medium, such as biological tissue, is governed by the Radiative Transfer Equation (RTE) [16]. However, the complexity of the RTE often requires approximations to make the problem computationally feasible. Common approaches include the spherical harmonics approximation, which leads to the SDA, and the δP1 model, which refines the SDA by incorporating the delta-Eddington approximation for the scattering phase function. These models rely on tissue optical properties, such as the absorption coefficient, scattering coefficient, and phase function, all of which are wavelength-dependent. By simplifying the RTE, these approximations enable practical calculations for applications like photothermal therapy. This section outlines the mathematical formulations of studied light and heat transport models, illustrating their utility in accurately describing the interactions between light, nanoparticles, and tissue.

### 2.1. Light Transport

The delta-Eddington approximation to the phase function further simplifies the P1 model, resulting in the δP1 model. These models rely on the optical properties of the material, including the absorption coefficient, scattering coefficient, and phase function, which vary with wavelength [16].

#### 2.1.1. Standard Diffusion Model

The SDA describes the physical behavior of the fluence rate, ϕ (Wm^−2^), using Equation (1), where $\mu_{a}$ is the absorption coefficient (m^−1^). The diffusion coefficient *D* is given by Equation (2) [22], and the reduced scattering coefficient $\mu_{s}^{*}$ is given by Equation (3), where *g* is the anisotropy factor.

(1) $$-D\nabla^{2}\phi +\mu_{a}\phi =S,$$

(2) $$D=\frac{1}{3\left(\mu_{a}+\mu_{s}^{*}\right)},$$

(3) $$\mu_{s}^{*}=\left(1-g\right)\mu_{s}.$$

Laser in the SDA is modeled as a Robin diffuse fluence rate boundary source, given by [23]

(4) $${\left.2AD\frac{\partial \phi }{\partial \vec{n}}\right|}_{\partial \Omega }=4P_{0}\left(r\right)-\phi ,$$

where $P_{0}\left(r\right)$ is the laser irradiance function on the surface (Wm^−2^) on the irradiated surface and A is a coefficient that describes radiation diverted due to Fresnel reflection [15]. Calculating this coefficient leads to the approximation of the Fresnel reflection moments $R_{1}$ and $R_{2}$. Using numerical integration on a normal slab for evaluation, the coefficient has been fitted to a cubic polynomial, given by Equation (5) [16].

(5) $$A=-0.13755n^{3}+43390n^{2}-4.09466n+168960,$$

where *n* is the ratio between the air and tissue refraction index.

#### 2.1.2. δP1 Model

The δP1 approximation simplifies the RTE by representing the phase function, which describes the angular scattering distribution as a delta function. This approach allows for the separation of collimated and diffuse radiation components. The resulting simplified equation can be further reduced to a diffusion equation [16]. Expanding the diffuse radiance of the RTE in Legendre polynomials leads to Equation (6).

(6) $$\nabla^{2}\phi_{d}-\mu_{eff}^{2}\phi_{d}=-3\mu_{s}^{*}\left(\mu_{tr}+\mu_{t}^{*}g^{*}\right)\phi_{c},$$

where $\phi_{d}$ and $\phi_{c}$ represent the diffuse and collimated components of the fluence rate $\left(W\;m^{-2}\right)$. The effective attenuation coefficient is defined as $\mu_{\mathrm{eff}}=\sqrt{3\mu_{a}\mu_{\mathrm{tr}}}$. The transport coefficient is $\mu_{\mathrm{tr}}=\mu_{a}+\mu_{s}^{\prime }$, where $\mu_{s}^{\prime }$ represents the isotropic scattering coefficient, and $\mu_{t}^{*}=\mu_{a}+\mu_{s}^{*}$, all with units of $\left(m^{-1}\right)$ [15]. According to Prahl, $\phi_{c}$ can be obtained by numerically solving [16]

(7) $$\frac{\partial \phi_{c}}{\partial \hat{s_{0}}}=-\mu_{t}^{*}\left(\vec{s}\right)\phi_{c},$$

where $\hat{s_{0}}$ represents the direction of collimated radiation. This equation, in particular, leads to the BL model on its own, which is a simplified model that neglects scattering events and is primarily applicable to purely absorbing media. The initial condition of this equation corresponds to the value of irradiance on the surface at a given point. Recalling that the phase function splits radiation into collimated and diffuse components, it is proposed that the first two moments of the phase function are functions of the fraction of light scattered forward. The first moment, *g*, is defined as the square of this fraction, while the second moment $g^{*}$ is given by $\frac{g}{1+g}$ [15]. When diffuse light is incident on a slab with different refraction index, it is required conservation of the diffuse flux component normal to the surface, which is given by [15].

(8) $${\left.\left(\phi_{d}-Ah\frac{\partial \phi_{d}}{\partial \vec{n}}\right)\right|}_{\partial \Omega }=-3Ahg^{*}\mu_{s}^{*}\phi_{c},$$

where *h* is given by Equation (9).

(9) $$h=\frac{2}{3\mu_{tr}}.$$

By solving Equations (6) and (7) subject to Equation (8), $\phi_{c}$ and $\phi_{d}$ are summed to compute the total fluence rate, which is then inserted to the bioheat equation as a source term.

### 2.2. Heat Transport Model

The Pennes bioheat equation obtained from an internal energy balance in a tissue is given by [6]

(10) $$\rho C_{p}\frac{\partial T}{\partial t}=k\nabla^{2}T+Q_{m}+Q_{b}+\mu_{a}\phi ,$$

where $C_{P}$ is the specific heat of the tissue, $Q_{m}$ is the heat generated by metabolic processes, $Q_{b}$ represents the heat exchange between blood and tissue due to perfusion, which can be expanded as $Q_{b}=W_{b}C_{b}\left(T-T_{a}\right)$ where $W_{b}$ is the blood perfusion rate indicating how much blood flows through the tissue, $C_{b}$ is the specific heat of blood describing how much heat the blood can carry and $T_{a}$ is the blood temperature [24]. $\mu_{a}\phi$ is the heat generated by nanoparticles. ϕ is the fluence rate calculated from the light transport model. When a tissue is exposed to ambient temperature, a convective heat flux arises due to the temperature difference at the interface. This flux is represented by a Robin boundary condition, given by [24].

(11) $${\left.-k\frac{\partial T}{\partial \vec{n}}\right|}_{\partial \Omega }=h\left(T-T_{ref}\right).$$

### 2.3. Numerical Implementation

This section outlines the numerical methods used to solve governing equations for light and heat transport in tissue. The SDA and δP1 models were implemented using the finite element method (FEM), with variational formulations adapted for cylindrical coordinates. MC simulations served as a benchmark for validating fluence distributions. For heat transport, the transient behavior was modeled using the bioheat equation, employing an implicit time-stepping algorithm and FEM spatial discretization.

#### 2.3.1. Monte Carlo Simulations

The Monte Carlo (MC) method was implemented using MCX from Monte Carlo Studio [25]. The geometry was constructed using basic shapes in the volume designer, with dimensions matching the validation case. A voxel size of 0.125 mm was employed for the simulations, utilizing 10^8^ photons [10]. The fluence rate was integrated and scaled over a time span of 1 s to achieve steady-state behavior for the fluence. Subsequently, the results were interpolated to the degrees of freedom (DOFs) of the FEM mesh using linear interpolation.

#### 2.3.2. Heat Transport Simulations

To obtain the transient temperature behavior governed by Equation (10), the model was expressed in cylindrical coordinates and spatially discretized using FEniCSx [26,27,28]. A backward Euler implicit scheme was employed for time stepping. The corresponding variational form is

(12) $$\begin{array}{cc} \int_{\Omega }\rho \;C_{P}\;T^{j+1}\;v\;r\;dV+\int_{\Omega }dt\;k\nabla T\nabla v\;r\;dV\; & +\int_{d\Omega }dt\;h\;T^{j+1}\;v\;r\;dS\\ \; & =\int_{\Omega }\rho \;C_{P}\;T^{j}\;v\;r\;dV+\int_{\Omega }Q_{S}\;v\;r\;dV\\ \; & \;+\int_{d\Omega }dt\;h\;T_{ref}\;v\;r\;dS. \end{array}$$

where $T^{j+1}$ and $T^{j}$ stand for future and old solutions respectively. The resulting linear system for each time step was solved using PETSc BigSTAB iterative solver [29].

#### 2.3.3. SDA and δP1 Simulations

Equations (1) and (8) were expressed in cylindrical coordinates, discretized, and solved numerically using the Finite Element method (FEM) implemented within the open-source FEniCSx framework, which comprises DOLFINx, basix, and UFL [26,27,28]. A variational formulation of the equations was derived using the Galerkin method with a first-order Lagrange function space. For the SDA model, the laser boundary condition was modeled using Equation (4). The resulting linear system was solved using the Portable, Extensible Toolkit for Scientific Computation (PETSc) LU decomposition direct solver [29]. For the δP1 model, Equation (7) was solved for each degree of freedom (DOF) of a DOLFINx function defined on the mesh, employing the fourth-order Runge–Kutta method provided by the SciPy solve_ivp function with the Python 3.13 framework [30]. Initial values for the fluence rate were assigned to the corresponding surface points of each DOF. The fluence rate distribution on the surfaces was varied depending on the specific case, accounting for both flat and Gaussian beam profiles. The resulting fluence rate distribution served as the source term in the solution of Equation (6).

## 3. Results and Discussion

This section presents validation studies and case simulations. The δP1 model was validated against analytical solutions provided by Carp et al. [15], comparing fluence rate distributions in semi-infinite media under Gaussian irradiation. Furthermore, a hypothetical case provided by Soni et al. simulating a skin tumor scenario with uniformly distributed gold nanorods (GNRs) was investigated, comparing the traditional diffusion and BL models with the δP1 approximation [7]. An additional case, derived from the experimental study by Lopes et al., simulated a gel phantom containing embedded GNRs subjected to Gaussian beam irradiation, with validation performed against experimental measurements [10].

### 3.1. δP1 Validation

Carp et al. [15] performed a series of case studies to evaluate the performance of the δP1 model against the established MC method. Their primary focus was on developing and assessing analytical solutions to characterize the fluence rate within a homogeneous, turbid, semi-infinite medium subjected to irradiation, considering both planar (1D) and Gaussian beam (2D) irradiation configurations. As our interest lies in 2D scenarios, we use a semi-infinite medium of length 100 $\mu_{tr}^{-1}$ ($l^{*}$). In the investigated cases, various values of laser waist ($r_{0}$), defined as the distance at which the intensity diminishes by a factor of $e^{-2}$, were compared to $l^{*}$. Values ranging from 1 to 100 $l^{*}$ were considered for this comparison. The results obtained through FEM simulations with boundary conditions given by the Equation (8) and P1−element distcretization were compared with the analytical solutions reported by the authors for $(\mu_{s}^{\prime }/\mu_{a})=100$. Note that $\phi_{d}$ boundary condition is a function of $\phi_{c}$, which is given by laser irradiation on the upper boundary and by the solution of Equation (7) in the rest of boundaries.

**Figure 1:**
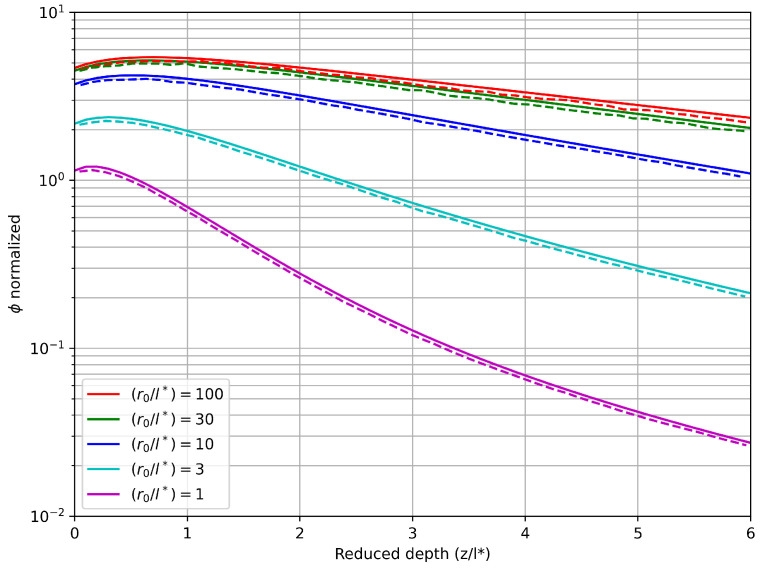
Numerical results of discretized δP1 model (–) against analytical results provided by Carp et al. (- -) [15].

Figure 1 presents the results of the δP1 model discretization for a case where the ratio between the laser waist and reduced depth is varied. A strong agreement is observed between the values obtained and those proposed by Carp et al. using analytical methods [15]. However, subtle discrepancies are evident. This divergence arises from the fact that the analytical solutions derived by the authors are applicable to semi-infinite media, while the mesh employed to discretize the equation is not sufficiently extensive to mitigate boundary effects, which become significant when viewed on a logarithmic scale. However, agreement on trends and values validates the methodology implemented to solve the δP1 model for a wide range of medium optical properties.

### 3.2. Case I—Heterogeneous Tissue Irradiated by a Flat Beam

**Figure 2:**
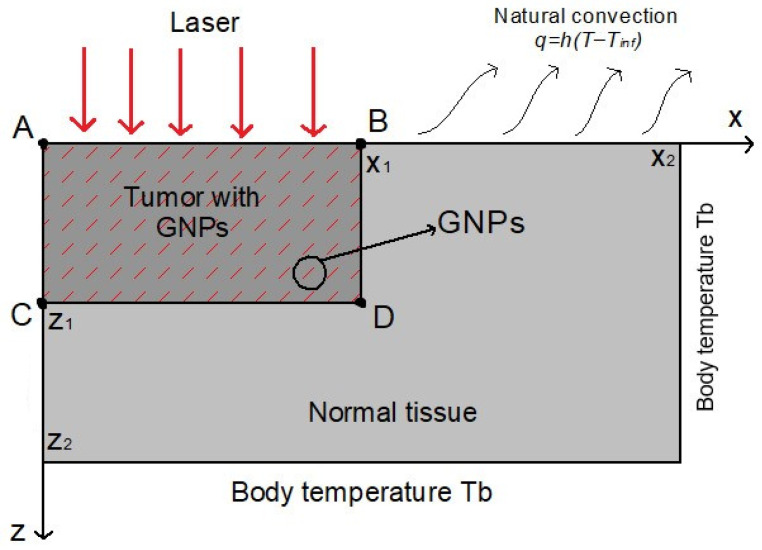
Geometry of a case I: Irradiation of heterogeneous tissue by a flat beam [7].

This case represents an idealized benchmark geometry used to isolate model behavior under controlled conditions for verifying fluence and temperature solution procedures. Soni et al. [7] provided a hypothetical scenario with a typical skin tumor using cylindrical geometry in two-dimensional coordinates. The tumor has a diameter of 20 mm and a depth of 5 mm, representing the dimensions of high-risk skin cancers. Surrounding the tumor is healthy tissue with a diameter of 40 mm and a depth of 10 mm, as shown in Figure 2.

**Figure 3:**
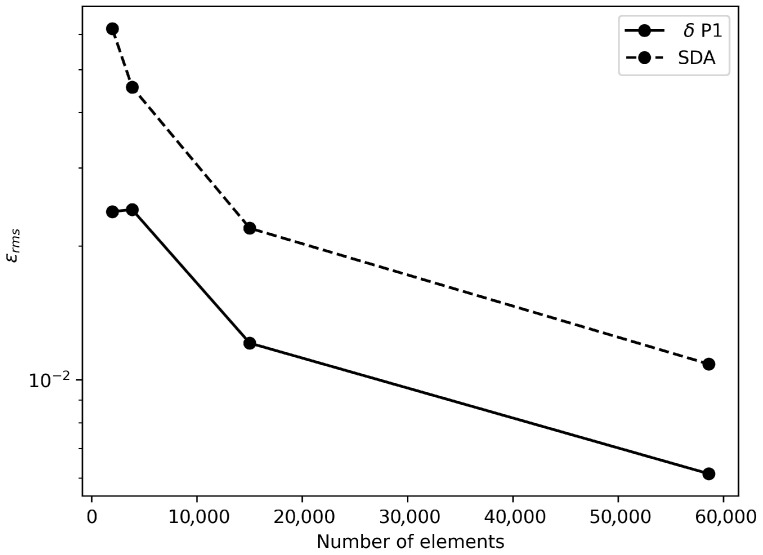
Fluence rate RMS error in terms of mesh size.

Fluence rate FEM results are obtained with equivalent boundary conditions as the validation case in Section 3.1 while temperature solution is achieved by imposing the convection condition (11) on the upper boundary, body temperature Dirichlet condition on right and bottom boundaries, and symmetry condition on left boundary. After the convergence study shown in Figure 3, a mesh of 15,008 triangular P1−elements was selected for spatial discretization of δP1 and bioheat equations. For solving the latter, the time step was fixed at 1 s as a suitable value to have a stable transient behavior. Optical and thermal properties for tumor and normal tissues reported in [7] are employed. Gold nanorods (GNRs) were considered for this investigation. For tumors situated near the surface, direct injection of nanoparticles can be a more practical approach, resulting in localized nanoparticle accumulation around the injection site [7]. In this specific case, GNRs were assumed to be distributed uniformly throughout the tumor region. This uniform distribution represents an idealized scenario. Although achieving such a distribution in practice is challenging, this case serves as a benchmark for comparison with other, more realistic non-uniform distributions.

**Figure 4:**
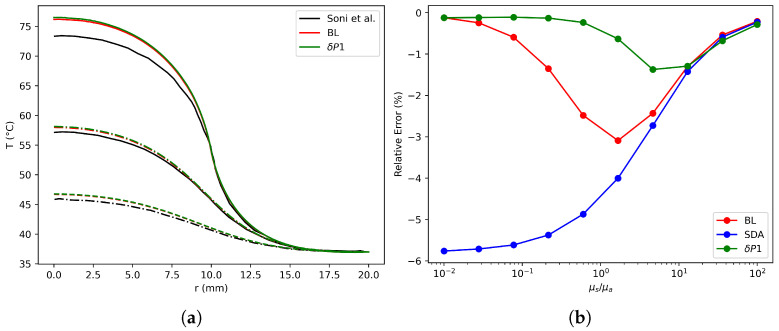
(**a**) Radial variation of temperature at different depths of validation case with different models [7]. (**b**) Models’ relative error analyses with varying optical coefficients [10].

With the aim of quantifying accuracy for δ-P1 and SDA models in comparison to MC solutions, RMS error for fluence rate spatial distribution is computed and shown in Figure 3. As the number of elements is increased, the error is reduced as expected for both methods with a similar rate. However, an improvement is obtained with the δ-P1 model since values are smaller for all mesh sizes. Furthermore, since the baseline case assumes the validity of the BL model, results were obtained using both the BL model and the δP1 approximation. The findings revealed acceptable concordance between the results reported by Soni et al. and those derived in this study. Moreover, the results exhibited excellent agreement between the BL model and the δP1 approximation, as depicted in Figure 4b.

To better represent realistic optical properties encountered in therapeutic applications, a new case was derived from the baseline scenario by reducing the extinction coefficient by an order of magnitude. Jain et al. [31] have previously demonstrated that variations in nanoparticle morphology and size significantly impact the relationship between absorption and scattering coefficients. Based on this knowledge, we analyzed the relative error of the model compared to MC across a range of $\mu_{a}/\mu_{s}$ ratios (presented in Figure 4b). The analysis revealed that the SDA model only exhibits reduced error under highly scattering conditions. While the BL model is valid primarily in scenarios with high absorption, its accuracy diminishes as scattering increases. Conversely, the δP1 approximation demonstrates superior performance by combining the strengths of both SDA and BL. This allows for accurate results across a broader spectrum, encompassing both high-scattering and high-absorbing regimes, while remaining reliable for intermediate optical properties typical of tissues containing nanoparticles.

### 3.3. Case II—Deep Tumor Irradiated by a Gaussian Beam

**Figure 5:**
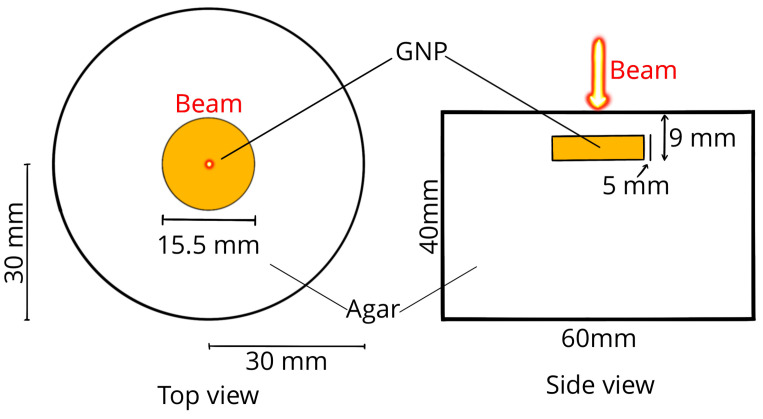
Case II geometry: A deep tumor is irradiated by a Gaussian beam.

This case corresponds to an experimental phantom designed to assess model performance under realistic deep-tissue irradiation conditions. Unlike case I, no metabolic and perfusion terms are included in the solution and numerical results are compared with experimental measurements. The conditions proposed by Lopes et al. [10] were modeled using the modified geometry depicted in Figure 5. The authors designed an agar gel phantom that incorporates embedded gold nanoparticles within a cylindrical subdomain. This gel was irradiated with a Gaussian beam characterized by a full-width at half-maximum (FWHM) of 7 mm and a power of 1.1 W [10]. The top surface of the domain was modeled using a Robin boundary condition, as defined by Equation (11), with a convection coefficient of 5 WK^−1^m^−2^. The remaining boundaries were assumed to be zero-flux conditions [10]. The optical properties of the domains, as determined by the authors, are presented in Table 1. Other properties, such as refractive index, specific heat, density, and thermal conductivity, were considered identical to those of water. The gel phantom was irradiated for a duration of 706 s. Again, after an independence study, a mesh of 17,241 triangular P1−elements was selected for spatial discretization of δP1 and bioheat equations. A time step of 2 s was fixed to solve the latter.

**Figure 6:**
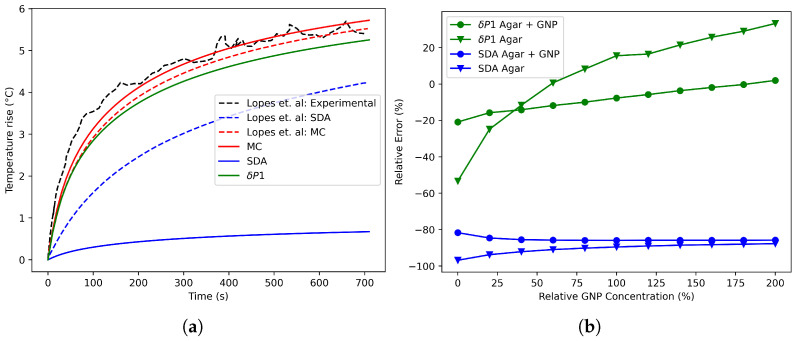
(**a**) Transient behavior of temperature at 4 mm depth of validation case with different models [10]. (**b**) Models’ relative error analyses in different regions varying relative GNP concentration.

The experimental configuration proposed by Lopes et al. is particularly well suited for the numerical implementation of photothermal therapy (PTT), as tumor irradiation often targets deep tissues. This approach ensures that therapy is delivered while minimizing its invasive effects [10]. To verify the correct application of the current model, the results reported by Lopes et al. were employed as reference values for validation purposes in deep tissues [10]. The results and experimental data obtained from the authors are presented in Figure 6b. The agreement between the experimental results and the proposed models indicates the reliability of the δP1 model, as the predicted temperatures closely align with the measurements reported and the MC results. However, certain discrepancies observed in the MC curve may be attributed to voxel resolution and uncertainties regarding the boundary conditions of the MC.

Beyond the absorption-to-scattering ratio explored in the previous case, PTT is also significantly influenced by the total extinction coefficient, which is directly related to the NP concentration. Maintaining accuracy across varying concentrations is crucial for accurate treatment modeling. Soni et al. [7] proposed a linear relationship between optical coefficients and concentration. Based on this finding, a linear model was used to correlate the optical properties of Table 1. The gold nanoparticle (GNP) region was designated as the reference point with a relative concentration of 100%, as specified by the experimental authors.

In PTT, it is essential to consider not only the average temperature within the tumor but also the temperature distribution in the surrounding tissue [32]. Therefore, the average temperature fields obtained with the SDA and the δP1 model were compared between different regions at the 706 s mark. These comparisons were performed against MC simulations for various relative GNP concentrations, considering that MC is a numerical reference-level solution and, in consequence, the objective of the deltaP1 model is not to surpass MC accuracy, but to achieve comparable accuracy at a reduced computational cost. The average temperature was obtained as

(13) $$T_{av}=\frac{\int_{\Omega_{R}}T\;r\;dV}{\int_{\Omega_{R}}r\;dV},$$

with $\Omega_{R}$ as the subdomain of integration.

Figure 6b presents a comparison of the errors between the models. The δP1 model demonstrates superior performance to the SDA model across the concentration spectrum analyzed. In particular, the δP1 model exhibits accurate results both within and outside the GNP region. The maximum observed temperature difference was 0.6 °C. The relative error in temperature was calculated by comparing the temperature predictions from the δP1 model with the corresponding reference data, as depicted in Equation (14).

(14) $$RE=\frac{|T_{\mathrm{model}}-T_{\mathrm{reference}}|}{T_{\mathrm{reference}}}\times 100.$$

This enhanced performance can be attributed to the δP1 model’s ability to independently propagate and scatter light, enabling greater generalization in forward-scattering media such as tissues [33]. However, despite its superiority over SDA, significant differences are observed in the Agar region, where lower temperature rises make it more susceptible to errors. Overall, the results indicate a wide range of GNP concentrations for which the model remains valid, even in heterogeneous and deep tissue scenarios, as proposed in the study case, which closely resembles realistic therapy geometries.

## 4. Conclusions

This study successfully validated the δP1 finite element implementation as a robust numerical method for simulating light propagation and thermal effects in biological tissues, particularly in the context of photothermal therapy (PTT). The model demonstrated superior performance compared to simpler models, such as the SDA and BL models, especially in domains with nanoparticles. The δP1 model accurately predicted temperature distributions in both homogeneous and heterogeneous tissue geometries, as evidenced by comparisons with Monte Carlo solutions and experimental data. Moreover, the model effectively handled heterogeneous geometries, such as those encountered in PTT. The model demonstrated robustness across a wide range of optical properties, including varying absorption and scattering coefficients, as well as different nanoparticle concentrations. These results highlight the potential of the δP1 approximation to significantly advance the field of photothermal therapy. By providing accurate predictions of light propagation and thermal effects, this model can aid in the development of more effective and personalized treatments. Future work will focus on the simulation and analysis of more realistic situations, including ex vivo, in vivo, and clinical conditions and geometries, to further refine the scope of the present work.

## Figures and Tables

**Table 1:** Optical properties measured in subdomains from Lopes et al. [10].

Property	Gel	GNP
$\mu_{a}$ (m^−1^)	2	31
$\mu_{s}$ (m^−1^)	176	289

## Data Availability

The raw data supporting the conclusions of this article will be made available by the authors on request.

## Abbreviations

The following abbreviations are used in this manuscript:

PTT	Photothermal therapy
RTE	Radiative transfer equation
BL	Beer–Lambert law
MC	Monte Carlo method
SDA	Standard diffusion approximation
FEM	Finite Element method
DOF	Degrees of freedom
FWHM	Full-width at half-maximum
GNR	Gold nanorods
